# Network meta-analysis with dose-response relationships

**DOI:** 10.1186/s12874-025-02754-4

**Published:** 2026-01-13

**Authors:** Maria Petropoulou, Gerta Rücker, Guido Schwarzer

**Affiliations:** Institute of Medical Biometry and Statistics, Faculty of Medicine and Medical Center - University of Freiburg, Freiburg, Germany

**Keywords:** Dose-response relationship, Network meta-analysis, Fractional polynomials, Restricted cubic splines

## Abstract

**Background:**

Network meta-analysis (NMA) is a widely used method for synthesizing evidence from multiple interventions for a medical condition. However, NMA applications typically ignore the crucial role of drug dosage on intervention effects. Traditional NMAs either consider each intervention dose as an independent node or ignore the intervention dose, which may impact heterogeneity, inconsistency, or sparsity.

**Methods:**

This paper introduces a novel frequentist approach, termed dose-response network meta-analysis (DR-NMA), which explicitly models the dose-response relationships across multiple interventions. The DR-NMA approach incorporates both linear and nonlinear dose-response relationships, including exponential, quadratic, fractional polynomials, and restricted cubic splines. DR-NMA allows for dose-dependent estimation and prediction of treatment effects across dose ranges, even in disconnected networks if common agents exist. The proposed methods are implemented in the R package netdose, enhancing accessibility and reproducibility. We illustrate the approach using clinical datasets on postoperative nausea and vomiting, as well as antidepressant treatments.

**Results:**

Our findings indicate that some dose-response NMA models yield substantially different results compared to standard NMA, emphasizing the critical importance of dose-response function selection in model performance.

**Conclusions:**

DR-NMA provides valuable insights into the dose-dependent effects of interventions, enhancing decision-making and offering perspectives beyond traditional methods.

**Supplementary Information:**

The online version contains supplementary material available at 10.1186/s12874-025-02754-4.

## Introduction

Pairwise meta-analysis is a statistical method that synthesizes evidence from studies to estimate the relative effect between two interventions, such as an active intervention (A) versus a placebo (P) [1]. Network meta-analysis (NMA) extends pairwise meta-analysis by allowing comparisons among more than two interventions, enabling the estimation of indirect intervention effects [2, 3].

Healthcare interventions often vary in aspects such as drug dosage or the number of sessions in cognitive behavioral therapy. In NMA, such interventions can be analyzed either separately (splitting approach) or as a combined intervention ignoring the dose (lumping approach) [1]. Under the splitting approach, each intervention dose is treated as having its own distinct effect. For example, consider a scenario that compares three active interventions with a placebo (P): intervention A and two different doses of intervention C (40 mcg or 60 mcg; mcg: micrograms). In the splitting approach, the different doses of intervention C ($C_{40mcg}$, $C_{60mcg}$) are treated as separate, unrelated nodes, resulting in four intervention nodes. Alternatively, in the lumping approach, the doses of intervention C are grouped together, resulting in three intervention nodes (A, C, and P).

Almost half of the NMAs published up to April 2015 report information on doses, typically using the lumping or splitting approach in the analysis [4]. Consequently, standard NMA applications typically ignore the impact of intervention dosage on treatment efficacy and safety. Modeling dose effects can address the limitations of lumping and splitting approaches, providing valuable insights for decision-making, informing future study designs, and supporting drug development. For decision-makers, this evaluation may identify not only the most effective intervention in a network but also the optimal dose.

Frequentist methods for pairwise dose-response meta-analysis are well established [5–11]. These methods integrate pairwise meta-analysis with models of dose-response relationships, capturing plausible pharmacological or biological interactions between responses and intervention doses. However, to our knowledge, no frequentist methods have been developed for dose-response NMA (DR-NMA).

Mandema et al. (2005) [12] first introduced a model-based network meta-analysis with dose–response relationships using the NONMEM software, a maximum-likelihood estimation method widely used in pharmacometrics. Subsequently, Bayesian approaches, including linear and Emax dose–response functions, have been developed for modeling dose–response relationships [13] and time-course effects [14]. More flexible approaches, such as restricted cubic splines (RCS), have recently been proposed for Bayesian meta-analysis [15] and NMA [16]. The Bayesian DR-NMA model has also been studied for its ability to reconnect disconnected networks if common agents link subnetworks [17]. Fractional polynomials have been used in individual participant data meta-analyses to explore effect modification by patient characteristics [18].

This paper introduces a frequentist approach to DR-NMA using fractional polynomials and restricted cubic splines. The proposed DR-NMA model is implemented in the open-source R package netdose [19]. The paper is organized as follows: Data section presents the motivating examples. In Methods section, we outline the standard frequentist NMA model (The standard NMA model section) and introduce the DR-NMA model (The dose-response NMA model section), addressing linear (Linear dose-response function section) and nonlinear dose-response relationships (Exponential dose-response function to Restricted cubic splines sections). The results of the examples are presented in Results section, followed by a discussion in Discussion section and the conclusions.

## Data

**Fig. 1:**
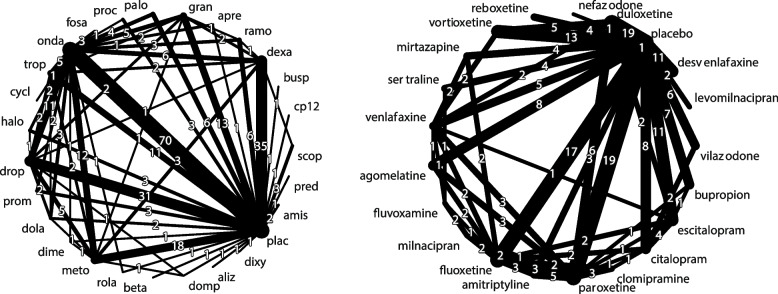
Network plot at the agent level. Left panel: Interventions to prevent post-operative nausea and vomiting; Right panel: Antidepressant interventions for unipolar major depressive disorder. Line widths correspond to the number of studies contributing to a comparison. Abbreviations for postoperative nausea and vomiting agents: aliz: alizapride; amis: amisulpride; apre: aprepitant; beta: betamethasone; busp: buspirone; cp12: CP-122,721; cycl: cyclizine; dexa: dexamethasone; dime: dimenhydrinate; dixy: dixyrazine; dola: dolasetron; domp: domperidone; drop: droperidol; fosa: fosaprepitant; gran: granisetron; halo: haloperidol; meto: metoclopramide; onda: ondansetron; palo: palonosetron; pred: prednisone; proc: prochlorperazine; prom: promethazine; plac: placebo; ramo: ramosetron; rola: rolapitant; scop: scopolamine; trop: tropisetron

The first dataset analyzed is derived from a published Cochrane review comprising 282 randomized controlled trials (RCTs) comparing interventions for the prevention of postoperative vomiting within 24 hours in adults undergoing general anesthesia [20, 21]. We considered a subset of the data in our analysis, excluding 80 studies evaluating multicomponent interventions and 3 studies lacking available dose data. The resulting dataset consists of 199 RCTs and 310 pairwise comparisons involving 27 agents and 86 interventions (i.e., agent and dose combinations). The effect measure used was the risk ratio (RR). These data are available in the R package netdose [19]. Figure 1 (left panel) depicts the network plot at the agent level.

The second dataset contains 170 RCTs evaluating the efficacy of antidepressants for unipolar major depressive disorder [22]. This dataset includes 21 antidepressants and placebo, using the odds ratio (OR) as the effect measure and the response rate as the primary outcome, defined as the proportion of patients achieving at least a 50% reduction in symptoms. Figure 1 (right panel) shows the network plot at the agent level for this dataset.

The antidepressant dataset has been analyzed previously using pairwise dose-response meta-analyses in both frequentist and Bayesian frameworks, as well as Bayesian DR-NMA with restricted cubic splines to model nonlinear dose-response relationships [23–25]. The general anesthesia example highlights real-world challenges with sparse dose levels (1 to 3 per agent; for most of the agents). The antidepressant example, with 3 to 12 dose levels per agent, provides a richer structure to model dose-response relationships, demonstrating the flexibility of DR-NMA in diverse data scenarios.

## Methods

**Fig. 2:**
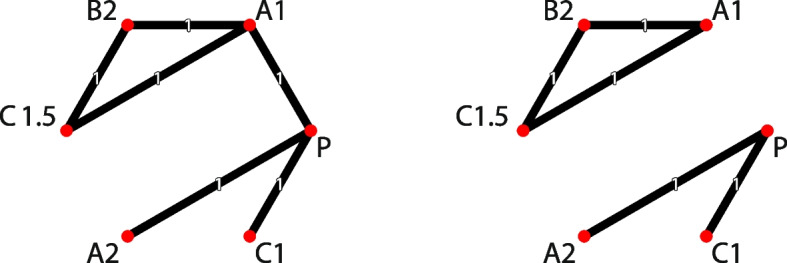
Network plot for the hypothetical example. Left panel: A connected network including the 6 studies; Right panel: A disconnected network created by excluding the first study

To introduce the DR-NMA model, we consider a hypothetical example involving $a = 4$ intervention agents: *A*, *B*, *C*, and placebo (*P*). The example includes $n = 6$ interventions: $A_1$, $A_2$, $B_2$, $C_1$, $C_{1.5}$, and *P*, and $m = 6$ pairwise comparisons/studies: $A_1$ - *P*, $A_1$ - $B_2$, $A_1$ - $C_{1.5}$, $B_2$ - $C_{1.5}$, $C_1$ - *P*, and $A_2$ - *P* (Table 1, Fig. 2 (left panel)). In this example, doses were standardized with respect to an agent-specific common dose: 20 mcg for agents *A* and *B*, and 40 mcg for agent *C*. Placebo *P* is assumed to be inactive, corresponding to 0 mcg (mcg: microgram). For example, a standardized dose of 1 for agent *A* corresponds to 20 mcg, whereas 1.5 for agent *C* corresponds to 60 mcg.

**Table 1:** Hypothetical data

Study	Arm 1		Arm 2		Intervention effect	Standard error
	Intervention	Dose	Intervention	Dose		
Study 1	*A*	1	*P*	0	$y_{1}$	$SE(y_{1})$
Study 2	*A*	1	*B*	2	$y_{2}$	$SE(y_{2})$
Study 3	*A*	1	*C*	1.5	$y_{3}$	$SE(y_{3})$
Study 4	*B*	2	*C*	1.5	$y_{4}$	$SE(y_{4})$
Study 5	*C*	1	*P*	0	$y_{5}$	$SE(y_{5})$
Study 6	*A*	2	*P*	0	$y_{6}$	$SE(y_{6})$

$\textbf{y} = (y_{1}, y_{2}, ..., y_{6})$ denotes the observed (relative) intervention effects (contrasts). Common dose: 20 mcg for *A* and *B*; 40 mcg for *C*; 0 mcg for *P*. mcg: microgram

### The standard NMA model

Standard NMA assumes that each intervention has its own effect, represented as a distinct node in the network. We follow the frequentist approach introduced by Rücker (2012) [26]. Suppose we have data consisting of *m* pairwise comparisons and *n* interventions, and let $\boldsymbol{\theta }$ represent the *n* intervention-based (true) effects.

For each comparison, the observed (relative) intervention effects are denoted by $\textbf{y} = (y_{1}, y_{2}, ..., y_{m})$, with associated standard errors $SE(\textbf{y}) = (\sigma _{1}, \sigma _{2}, ..., \sigma _{m})$. Assuming a common between-study variance (heterogeneity $\tau ^2$) across the pairwise comparisons, the random-effects network meta-analysis model is given by:

1 $$\begin{aligned} \textbf{y} = \textbf{X}\boldsymbol{\theta } + \boldsymbol{\mu } + \boldsymbol{\epsilon }, \boldsymbol{\epsilon } \sim N(\textbf{0},\boldsymbol{\Sigma }), \boldsymbol{\mu }\sim N(\textbf{0},\boldsymbol{\Delta }) \end{aligned}$$

where $\textbf{X}$ is the design matrix with *m* rows denoting the pairwise comparisons and with *n* columns the interventions compared [26]. Each row of the design matrix $\textbf{X}$ places one (1) in the column corresponding to the first intervention and minus one $(-1)$ in the column corresponding to the second intervention. The matrix $\boldsymbol{\Sigma }$ represents the within-study (co)variance matrix for the sampling errors $\boldsymbol{\epsilon }$, and $\boldsymbol{\Delta }$ denotes the between-study (co)variance matrix for the true random effects $\boldsymbol{\mu }$.

Let $\textbf{W}$ be a diagonal $m \times m$ weight matrix, with a vector of weights along its diagonal. The weight for each two-arm study is the inverse of the sum of the within- and between-study variance estimators ($\hat{\tau }^2$). In multi-arm trials, observed pairwise comparisons share at least one common arm and are therefore correlated, so the weights are adjusted using the “reduced weights” approach described by Rücker and Schwarzer (2014) [27].

The standard NMA model can be expressed briefly as $\boldsymbol{\delta } = \textbf{X}\boldsymbol{\theta }$, where $\boldsymbol{\delta }$ is the vector of true relative intervention effects. Estimation of the true parameters is performed using weighted least squares regression:

2 $$\begin{aligned} \hat{\boldsymbol{\delta }} = \textbf{X}(\textbf{X}^\top \textbf{W} \textbf{X})^{+} \textbf{X}^\top \textbf{W} \textbf{y} = \textbf{H} \textbf{y}, \end{aligned}$$

with the estimated covariance matrix

$$\begin{aligned} \widehat{\text {Cov}}\,(\hat{\boldsymbol{\delta }}) = \textbf{X} (\textbf{X}^\top \textbf{W} \textbf{X})^{+} \textbf{X}^{\top }, \end{aligned}$$

where $\textbf{H}$ is the hat matrix that maps the observed effects $\textbf{y}$ to their model-based estimates: $\textbf{H} \textbf{y} = \hat{\boldsymbol{\delta }}$.

Cochran’s *Q* statistic is commonly used to assess statistical heterogeneity across studies. The *Q* statistic for the standard NMA is given by:

$$\begin{aligned} Q = (\textbf{y} - \hat{\boldsymbol{\delta }})^\top \textbf{W} (\textbf{y} - \hat{\boldsymbol{\delta }}) \end{aligned}$$

Further details about the NMA model are available in Rücker (2012) and Rücker et al. (2014) [26, 27].

For the hypothetical data in Table 1, the network structure is described by the design matrix $\textbf{X}$, given as:

$$\begin{aligned} \textbf{X} = \left( \begin{array}{cccc} 1 & 0 & 0 & -1 \\ 1 & -1 & 0 & 0 \\ 1 & 0 & -1 & 0 \\ 0 & 1 & -1 & 0 \\ 0 & 0 & 1 & -1 \\ 1 & 0 & 0 & -1 \\ \end{array}\right) \end{aligned}$$

where the columns correspond to the interventions *A*, *B*, *C*, and *P*.

### The dose-response NMA model

In the hypothetical example, intervention *C* has two doses (Table 1). A standard NMA would either treat these as one node (lumping approach) or two separate nodes (splitting approach), without considering the specific doses. In the DR-NMA model, we incorporate the doses into the analysis by extending the NMA model to account for a pharmacological/biological relationship between different doses and responses.

For *n* interventions and *a* intervention agents, each associated with observed doses $d_{ij}, \ i = 1, \dots , n; \ j = 1, \dots , a$, we construct the observed-dose matrix $\textbf{D}_{obs}$. This $n \times a$ matrix contains the dose entries $d_{ij}$ for each intervention in the network, positioned in the columns corresponding to the respective intervention agents, with zeros elsewhere.

#### Linear dose-response function

We begin by assuming a linear dose-response relationship. The linear DR-NMA model is expressed as:

3 $$\begin{aligned} \boldsymbol{\delta } = \textbf{X}_d\boldsymbol{\theta } \end{aligned}$$

where $\boldsymbol{\theta }$ represents the *a* agent effects, and $\boldsymbol{\delta }$ denotes the vector of *m* true relative agent effects. For simplicity, we use the same notation, $\boldsymbol{\theta }$ and $\boldsymbol{\delta }$, for both the standard NMA and DR-NMA models, including at the estimation stage (i.e., $\hat{\boldsymbol{\theta }}$ and $\hat{\boldsymbol{\delta }}$).

To define the model structure, we introduce two key matrices:

- The $m \times n$ **structure matrix** $\textbf{B}$, which has *m* rows corresponding to the pairwise comparisons and *n* columns representing the interventions. It describes the network structure by placing entries of 1 and -1 in the columns corresponding to the interventions being compared, with zeros elsewhere. This matrix corresponds to the edge-vertex incidence matrix as defined in Rücker (2012) [26].
- The $n \times a$ **dose-transformation matrix** $\textbf{D}$ is constructed by applying a transformation function *g* element-wise to the observed-dose matrix $\textbf{D}_{obs}$. For a linear dose-response relationship, the transformation function is the identity, i.e., $g(\textbf{D}_{obs}) = \textbf{D}_{obs} = \textbf{D}$. This implies that each entry of $\textbf{D}_{obs}$ is retained directly as the observed dose value, that is, $g(d_{ij}) = d_{ij}$.

The design matrix $\textbf{X}_{d}$ for the DR-NMA model is then obtained as:

4 $$\begin{aligned} \textbf{X}_{d} = \textbf{B} \textbf{D}. \end{aligned}$$

For the hypothetical data in Table 1, the linear dose-response function is applied to the intervention agents *A*, *B*, *C*, and *P*. The network structure is described by the structure matrix $\textbf{B}$:

$$\begin{aligned} \textbf{B} = \left( \begin{array}{cccccc} 1 & 0 & 0 & 0 & 0 & -1 \\ 1 & 0 & -1 & 0 & 0 & 0 \\ 1 & 0 & 0 & 0 & -1 & 0 \\ 0 & 0 & 1 & 0 & -1 & 0 \\ 0 & 0 & 0 & 1 & 0 & -1 \\ 0 & 1 & 0 & 0 & 0 & -1 \\ \end{array}\right) \end{aligned}$$

where the rows represent the $m=6$ pairwise comparisons, and the columns correspond to the interventions $A_{1}$, $A_{2}$, $B_{2}$, $C_{1}$, $C_{1.5}$, and *P*.

The dose-transformation matrix $\textbf{D}$ is given by:

$$\begin{aligned} \textbf{D} = \textbf{D}_{obs} = \left( \begin{array}{cccc} 1 & 0 & 0 & 0 \\ 2 & 0 & 0 & 0 \\ 0 & 2 & 0 & 0 \\ 0 & 0 & 1 & 0 \\ 0 & 0 & 1.5 & 0 \\ 0 & 0 & 0 & 0 \\ \end{array}\right) \end{aligned}$$

Here, the rows correspond to the interventions, and the columns correspond to the intervention agents *A*, *B*, *C*, and *P*. The last column represents the reference intervention agent (placebo) and contains all zeros (inactive intervention agent).

The resulting design matrix for the linear dose-response DR-NMA model is:

$$\begin{aligned} \textbf{X}_{d} = \textbf{B}\textbf{D} = \left( \begin{array}{cccc} 1 & 0 & 0 & 0 \\ 1 & -2 & 0 & 0 \\ 1 & 0 & -1.5 & 0 \\ 0 & 2 & -1.5 & 0 \\ 0 & 0 & 1 & 0 \\ 2 & 0 & 0 & 0 \\ \end{array}\right) \end{aligned}$$

The linear dose-response model is a first–degree polynomial model that often fails to adequately reflect complex dose–effect relationships in natural settings.

#### Exponential dose-response function

The exponential dose-response model provides an alternative approach that can more effectively capture nonlinear dose-response patterns. This model is particularly suitable for scenarios where the response variable demonstrates exponential growth or decay in relation to changes in dose levels [28].

The DR-NMA model based on the exponential transformation is expressed as in Eq. (3), where the $n \times a$ dose-transformation matrix is defined as

$$\begin{aligned} \textbf{D} = g(\textbf{D}_{\text {obs}}) = 1 - e^{-\lambda \textbf{D}_{\text {obs}}}, \end{aligned}$$

with $\lambda$ denoting a rate parameter. The transformation is applied element-wise to each observed dose, such that $g(d_{ij}) = 1 - e^{-\lambda d_{ij}}$.

In principle, $\lambda$ may be set to a simple fixed value (i.e., $\lambda = 1$). Alternatively, it can be chosen as a fixed scaling constant that controls the curvature of the transformed dose scale. For example, in settings with relatively homogeneous dose ranges, one may select $\lambda$ such that the largest observed dose maps to a prespecified value (e.g. 0.95),

$$\begin{aligned} \lambda = -\frac{\log (1 - 0.95)}{\max (d_{ij})}. \end{aligned}$$

In our analyses, we set $\lambda = 1$, a simple and commonly used choice that produces a monotonic transformation without additional tuning. Accordingly, for the hypothetical data in Table 1 and $\lambda =1$, the resulting dose-transformation matrix is:

$$\begin{aligned} \textbf{D} = \left( \begin{array}{cccc} 1-e^{-1} & 0 & 0 & 0 \\ 1-e^{-2} & 0 & 0 & 0 \\ 0 & 1-e^{-2} & 0 & 0 \\ 0 & 0 & 1-e^{-1} & 0 \\ 0 & 0 & 1-e^{-1.5} & 0 \\ 0 & 0 & 0 & 0 \\ \end{array}\right) \end{aligned}$$

Here, the rows correspond to the interventions, whereas the columns represent the intervention agents *A*, *B*, *C*, and *P*. The last column, which corresponds to the reference intervention agent (placebo), contains all zeros, indicating an inactive intervention agent.

#### Fractional polynomials

Traditional polynomials, such as quadratic (second-degree) and cubic (third-degree) polynomials, provide flexibility for modeling complex relationships, including dose-response curves. For example, a quadratic polynomial dose-response function extends the linear transformation by adding a term for the squared dose variable, while a cubic polynomial includes an additional term for the cubed dose variable. These terms allow the model to capture nonlinear relationships between dose and response, enabling U- or S-shaped patterns. Such patterns can highlight an optimal dose range where the intervention achieves maximum efficacy or safety. However, adding more terms to a polynomial increases flexibility, which can lead to overfitting, especially in small or noisy datasets. While quadratic and cubic polynomials can overfit, this risk is greater with higher-degree polynomials that have many terms.

Fractional polynomials provide a more flexible yet simpler approach to capture nonlinear relationships, especially in clinical research contexts [29, 30]. Jansen et al. incorporated fractional polynomials into a NMA model for outcomes with multiple time points [31, 32]. Fractional polynomials use powers with fractional exponents, allowing for a controlled level of flexibility without the potential overfitting issues associated with high-degree polynomials [30]. This makes them particularly valuable in dose-response modeling, where achieving a balance between model complexity and interpretability is crucial.

The DR-NMA model based on a fractional polynomial transformation of order 1 (FP1) can be expressed as in Eq. (3), where the $n \times a$ dose-transformation matrix $\textbf{D}$ is given by

$$\begin{aligned} \textbf{D} = g(\textbf{D}_{\text {obs}}) = \left\{ \begin{array}{ll} \log (\textbf{D}_{\text {obs}} + c), & \text {if}\ p = 0 \\ \textbf{D}_{\text {obs}}^p, & \text {if}\ p \ne 0 \end{array}\right. \end{aligned}$$

where $c$ is a small positive constant used to stabilize the logarithmic transformation near zero (i.e., 0.001). The predefined set of candidate values for *p*, denoted as $S = \{-2, -1, -0.5, 0, 0.5, 1, 2, 3\}$, provides a practical range to explore nonlinear relationships [29, 30]. The DR-NMA models are implemented for each candidate value of $p$ in the set $S$. Notably, when $p = 1$, the FP1 transformation simplifies to the linear dose-response relationship. The selection of the specific *p* value is often guided by model fit statistics [30, 33], with the Q-statistic being used here. This approach facilitates the identification of the most appropriate FP1 transformation for modeling the dose-response relationship.

For the hypothetical data in Table 1, the dose-transformation matrix $\textbf{D}$ for an FP1 with $p=2$ is given by:

$$\begin{aligned} \textbf{D} = \left( \begin{array}{cccc} 1 & 0 & 0 & 0 \\ 4 & 0 & 0 & 0 \\ 0 & 4 & 0 & 0 \\ 0 & 0 & 1 & 0 \\ 0 & 0 & 2.25 & 0 \\ 0 & 0 & 0 & 0 \\ \end{array}\right) \end{aligned}$$

This leads to the following design matrix:

$$\begin{aligned} \textbf{X}_{d} = \textbf{BD} = \left( \begin{array}{cccc} 1 & 0 & 0 & 0 \\ 1 & -4 & 0 & 0 \\ 1 & 0 & -2.25 & 0 \\ 0 & 4 & -2.25 & 0 \\ 0 & 0 & 1 & 0 \\ 4 & 0 & 0 & 0 \\ \end{array}\right) \end{aligned}$$

#### Restricted cubic splines

Splines provide flexibility in capturing nonlinear relationships. Among them, restricted cubic splines (RCS) form a family of smooth functions particularly well suited for modeling diverse dose-response relationships. The RCS consists of piecewise polynomials defined over consecutive intervals. The values defining these disjoint intervals are known as knots. This approach allows for the exploration of complex dose-response patterns, providing a balance between flexibility and the risk of overfitting.

In the context of an RCS model with three knots ($\textbf{k} = (k_1, k_2, k_3)$), the resulting DR-NMA model with two ($\textrm{length}(\textbf{k}) - 1$) regression coefficients is given by:

5 $$\begin{aligned} \boldsymbol{\delta } = \textbf{X}_{d_1}\boldsymbol{\theta }_1 + \textbf{X}_{d_2}\boldsymbol{\theta }_2 = \textbf{X}_d \boldsymbol{\theta } \end{aligned}$$

6 $$\begin{aligned} \textbf{X}_d = \left[ \textbf{X}_{d_1} \ |\ \textbf{X}_{d_2} \right] = \left[ \textbf{B} \textbf{D}_1\ |\ \textbf{B} \textbf{D}_2 \right] = \textbf{B} \left[ \textbf{D}_1\ |\ \textbf{D}_2 \right] \end{aligned}$$

where $\textbf{X}_d$ is the $m \times 2a$ design matrix for the DR-NMA model with RCS, obtained by concatenating two matrices: $\textbf{X}_{d_1} = \textbf{B} \textbf{D}_1$ and $\textbf{X}_{d_2} = \textbf{B} \textbf{D}_2$. Let $\boldsymbol{\theta }$ be the 2*a* concatenation vector of agent effects, expressed as $\boldsymbol{\theta } = (\boldsymbol{\theta }_1, \boldsymbol{\theta }_2)$, where $\boldsymbol{\theta }_1$ and $\boldsymbol{\theta }_2$ are the *a* agent effects for the first transformation ($g_1$) and the second transformation ($g_2$), respectively. The vector $\boldsymbol{\delta }$ is an *m*-dimensional vector containing the true relative agent effects obtained from the transformations $g_1$ and $g_2$.

The matrix $\textbf{B}$ is the $m \times n$ edge-vertex incidence matrix, as provided above. The matrices $\textbf{D}_{1}$ and $\textbf{D}_{2}$ are the $n \times a$ dose-transformation matrices for the transformations $g_{1}$ and $g_{2}$, respectively. The functions $g_{1}$ and $g_{2}$ are applied element-wise to each cell of the $n \times a$ observed dose matrix $\textbf{D}_{obs}$ to characterize the dose-response relationships, i.e., $\textbf{D}_{1} = g_{1}(\textbf{D}_{obs})$ and $\textbf{D}_{2} = g_{2}(\textbf{D}_{obs})$.

The RCS model with three knots, denoted by $\textbf{k} = (k_{1}, k_{2}, k_{3})$, involves two primary transformations: a linear transformation $g_1(x)$ and a restricted cubic spline transformation $g_2(x)$:

7 $$\begin{aligned} \textbf{D}_{1} = g_{1}(\textbf{D}_{obs}) = \textbf{D}_{obs} \end{aligned}$$

8 $$\begin{aligned} \textbf{D}_{2} = g_{2}(\textbf{D}_{obs}) = \frac{(\textbf{D}_{obs}-k_{1})^3_{+} - \frac{k_{3}-k_{1}}{k_{3}-k_{2}}(\textbf{D}_{obs}-k_{2})^3_{+} + \frac{k_{2}-k_{1}}{k_{3}-k_{2}}(\textbf{D}_{obs}-k_{3})^3_{+}}{(k_{3}-k_{1})^2} \end{aligned}$$

In Eq. (8), the “$(\cdot )_+$” notation indicates that $u_{+} = u$ if $u \ge 0$ and $u_{+} = 0$ otherwise [34]. For an inactive agent (e.g., placebo), the dose is set to zero, and the transformations $g_{1}(0) = 0$ and $g_{2}(0) = 0$ are applied. The RCS function is available in the R package **Hmisc** [35].

According to Stone (1986) [36], the number of knots is more crucial than their location for the model fit. For small samples (less than 30), a choice of three knots provides an adequate fit and serves as a good compromise between flexibility and the potential overfitting, which could lead to a loss of precision [36]. Harrell (2015) [34] recommends placing the knots at equally spaced quantiles, specifically at the 10th, 50th, and 90th percentiles. A sensitivity analysis can be conducted by varying the knot positions — for example, using alternative percentiles such as the 25th, 50th, and 75th percentiles — to assess the robustness of the dose-response estimates to knot placement. In our implementation, knots are computed separately for each agent by calculating the specified percentiles within the distribution of that agent’s observed doses. Further details about the RCS function, knot selection, and location can be found in Harrell (2015) [34].

For the hypothetical data in Table 1, we implement the RCS dose-response function for each intervention agent *A*, *B*, *C*, and *P*. To illustrate the RCS transformations, we use the 25th, 50th, and 75th percentiles to be computed separately within the observed dose distribution of each agent. The resulting agent-specific knots are:

$$\begin{aligned} \begin{array}{ll} \text {A:} & (1.25,\ 1.50,\ 1.75) \\ \text {B:} & (2,\ 2,\ 2) \\ \text {C:} & (1,\ 1,\ 1) \\ \text {P:} & (0,\ 0,\ 0) \end{array} \end{aligned}$$

Agents *B* and *C *have only one observed dose, and placebo P corresponds to dose zero. In these cases, the percentiles collapse to identical values, and an RCS curve is not identifiable. Thus,

- for agent *A*, both a linear and a non-linear spline term ($A_{1}, A_{2}$) can be constructed,
- for agents *B* and *C*, only linear dose terms ($B_{1}, C_{1}$) are feasible,
- for placebo *P*, no dose term is included (baseline).

In fact, RCS requires at least three distinct knots per agent; otherwise, the model must revert to simpler specifications (linear). In practice, richer dose-response data are required to obtain a meaningful RCS fit; this goes beyond the scope of our artificial dataset but is demonstrated in our real applied analyses.

#### Estimation and random-effects DR-NMA

Once the dose transformation is selected and the design matrix $\textbf{X}_d$ for the network has been constructed, the weighted least squares estimation for $\boldsymbol{\theta }$ can be performed. For simplicity, we begin with the common-effects DR-NMA model. The weight matrix $\textbf{W}$ is a $m \times m$ diagonal matrix containing the observed inverse variances within the study of all existing comparisons. In the case of multi-arm studies, standard errors are recalculated (increased) with an adjustment [27], $\sigma _{i,\text {adj}}^2$, and new reduced weights are derived.

Based on the theory for standard NMA [26, 27], the weighted least squares estimates of the agent effects $\boldsymbol{\theta }$ are given by:

9 $$\begin{aligned} \hat{\boldsymbol{\theta }} = (\textbf{X}_d^\top \textbf{W} \textbf{X}_d)^{+} \textbf{X}_d^\top \textbf{W} \textbf{y} \end{aligned}$$

with the estimated covariance matrix:

$$\begin{aligned} \widehat{\text {Cov}}\,(\hat{\boldsymbol{\theta }}) = (\textbf{X}_{d}^{\top } \textbf{W} \textbf{X}_d)^{+} \end{aligned}$$

The comparisons (contrasts) $\boldsymbol{\delta }$ are estimated by:

10 $$\begin{aligned} \hat{\boldsymbol{\delta }} = \textbf{X}_d \hat{\boldsymbol{\theta }} \end{aligned}$$

with covariance matrix:

$$\begin{aligned} \widehat{\text {Cov}}\,(\hat{\boldsymbol{\delta }}) = \textbf{X}_d (\textbf{X}_d^\top \textbf{W} \textbf{X}_d)^{+} \textbf{X}_d^\top . \end{aligned}$$

In the context of the DR-NMA model, Cochran’s *Q* statistic, denoted as $Q_d$, is defined as:

$$\begin{aligned} Q_d = (\textbf{y} - \hat{\boldsymbol{\delta }})^\top \textbf{W} (\textbf{y} - \hat{\boldsymbol{\delta }}) \end{aligned}$$

Conditional on the variances that are assumed to be known, this statistic follows a $\chi ^2$-distribution with degrees of freedom $df_d = n_a - k - r$, where $n_a$ is the total number of intervention arms, *k* is the number of studies, and *r* is the rank of the design matrix $\textbf{X}_d$. For two-arm trials, we obtain $n_a = 2k$, resulting in $df_d = k - r$ degrees of freedom. In the case of linear and fractional polynomial transformations of order 1, the rank of the design matrix $\textbf{X}_d$ is $r = a - 1$, yielding $df_d = n_a - k - (a - 1)$. For RCS transformations, the rank is $r = 2a - c$, where *c* is the number of constraints, including the reference agent and potential collinearity between the $\textbf{D}_{1}$ and $\textbf{D}_{2}$ matrices.

The ratio $Q_{d}/df_{d}$ provides an indicator of model adequacy relative to its complexity. The (DR-)NMA model with the lowest value is considered to achieve the best balance between fit and parsimony.

A random-effects DR-NMA model, assuming a common between-study variance $\tau ^2$, can be implemented analogously to the standard NMA approach [27], using a multivariate method-of-moments estimator [37]:

$$\begin{aligned} \hat{\tau }^2 = \max \left( \frac{Q_d - df_d}{\text {tr}\left( (\textbf{I} - \textbf{H}_d)\textbf{U}\textbf{W}\right) }, 0\right) \end{aligned}$$

where $Q_d$, $df_d$, and $\textbf{W}$ are defined above. $\textbf{I}$ is the $m \times m$ identity matrix, and $\text {tr}$ denotes the trace of a matrix (the sum of its diagonal elements). $\textbf{U}$ is a block diagonal matrix derived from the $m \times m$ matrix $0.5 \cdot \textbf{B} \textbf{B}^\top$, selecting each *j*-arm study’s $j \times j$ block and setting all other matrix elements to zero. $\textbf{H}_d$ is the hat matrix for the DR-NMA model, defined as:

$$\begin{aligned} \textbf{H}_d = \textbf{X}_d (\textbf{X}_d^\top \textbf{W} \textbf{X}_d)^{+} \textbf{X}_d^\top \textbf{W} \end{aligned}$$

The heterogeneity estimator $\hat{\tau }^{2}$ is added to the observed within-study variances $\sigma _i^2$ for each pairwise comparison. For multi-arm studies, the within-study variances are adjusted in the same way as in the common-effects model [27]. In the general case, the $m \times m$ weight matrix $\textbf{W}$ can be constructed with weight entries $w_i = 1 / (\hat{\tau }^{2} + \sigma _{i,\text {adj}}^2)$ on its diagonal, and the weighted least squares estimates can be computed.

#### DR-NMA for disconnected networks

In evidence synthesis, a disconnected network occurs when certain interventions cannot be compared directly or indirectly due to the absence of a common comparator or linking studies. This creates challenges for estimating relative treatment effects, as traditional NMA depends on network connectivity to conduct the analysis.

To illustrate this issue, we use a modified version of the hypothetical dataset. The network includes six interventions: $A_{1}$, $A_{2}$, $B_{2}$, $C_{1}$, $C_{1.5}$, and *P*, as presented in Table 1. If we remove the first study comparing $A_{1}$ with *P*, the network becomes disconnected, dividing it into two subnetworks (Fig. 2 (right panel)). One subnetwork comprises interventions $A_{2}$, $C_{1}$ and *P*, while the other consists of $A_{1}$, $B_{2}$, and $C_{1.5}$. In this scenario, the relative effects between intervention agents *A*, *B*, and *C* at any dose cannot be estimated using standard NMA due to lack of connectivity.

Several approaches have been suggested to address this issue. These include the use of real-world evidence [38], alternative Bayesian models [39], indirect population adjustment comparisons [40–42], a mixture of study designs [43], and modeling intervention components [44–47] to connect networks. The DR-NMA model can reconnect networks if subnetworks contain shared intervention agents. Pedder et al. (2021) [17] presented several scenarios illustrating how a DR-NMA model can reconnect disconnected networks and deliver more precise results than alternative approaches.

In our example, the DR-NMA model utilizes shared intervention agents present in different subnetworks to establish a connection and to model the dose-response relationship. To demonstrate this, we apply a linear dose-response relationship to our hypothetical dataset. The resulting design matrix is

$$\begin{aligned} \textbf{X}_{d} = \textbf{B} \textbf{D} &= \left( \begin{array}{cccccc} 1 & 0 & -1 & 0 & 0 & 0 \\ 1 & 0 & 0 & 0 & -1 & 0 \\ 0 & 0 & 1 & 0 & -1 & 0 \\ 0 & 0 & 0 & 1 & 0 & -1 \\ 0 & 1 & 0 & 0 & 0 & -1 \\ \end{array}\right) \left( \begin{array}{cccc} 1 & 0 & 0 & 0 \\ 2 & 0 & 0 & 0 \\ 0 & 2 & 0 & 0 \\ 0 & 0 & 1 & 0 \\ 0 & 0 & 1.5 & 0 \\ 0 & 0 & 0 & 0 \\ \end{array}\right) \\&= \left( \begin{array}{cccc} 1 & -2 & 0 & 0 \\ 1 & 0 & -1.5 & 0 \\ 0 & 2 & -1.5 & 0 \\ 0 & 0 & 1 & 0 \\ 2 & 0 & 0 & 0 \\ \end{array}\right) \end{aligned}$$

which has rank 3, provided that the intervention agents *A*, *B* and *C* can be uniquely estimated.

## Results

We implemented the standard NMA and several DR-NMA models with various dose-response functions, including linear, quadratic, exponential ($\lambda = 1$), and fractional polynomials (with the recommended set of powers), and an RCS with two knots placement scenarios. Analyses were conducted on the general anesthesia and antidepressant datasets described in Data section. DR-NMA models were fitted using the netdose() function from the R package netdose [19]. The corresponding code is provided in Additional file 1.

Below, we present detailed results for the standard NMA and DR-NMA models based on exponential, FP1, and RCS transformations for both datasets.

### Results of the general anesthesia dataset

Table 2 presents an overview of the performance of various (DR-)NMA models based on model adequacy and heterogeneity metrics. Among the DR-NMA models evaluated, the exponential model has the lowest ratio $Q_{d}/df_d = 2.11$, followed by FP1 with powers $p=0.5$ and $p=-0.5$ ($2.28$ and $2.76$, respectively), and the RCS model with knots at the 10th, 50th, and 90th percentiles ($Q_{d}/df_d =2.76$).

**Table 2:** (DR-)NMA ratio and heterogeneity metrics for the general anesthesia dataset (sorted by $Q_{\text {d}} / df_{\text {d}}$)

Model	*Q*_d_ (*df*_*d*_)	*Q*_d_/*df*_*d*_	*p*-value(*Q*_d_)	$\hat{\boldsymbol{\tau }}^{\boldsymbol{2}}$	*I*^2^ (%)
NMA	448.37 (225)	1.99	$< 0.0001$	0.0612	49.8
Exponential	475.02 (225)	2.11	$< 0.0001$	0.1578	52.6
FP1 ($p=0.5$)	512.79 (225)	2.28	$< 0.0001$	0.0229	56.1
FP1 ($p=-0.5$)	621.98 (225)	2.76	$< 0.0001$	0.1007	63.8
RCS (10/50/90%)	587.62 (213)	2.76	$< 0.0001$	0.0007	63.8
RCS (25/50/75%)	595.33 (213)	2.79	$< 0.0001$	0.0007	64.2
FP1 ($p=0$)	673.73 (225)	2.99	$< 0.0001$	0.0742	66.6
Quadratic	712.42 (211)	3.38	$< 0.0001$	$< 0.0001$	70.4
Linear/FP1 ($p=1$)	818.52 (225)	3.64	$< 0.0001$	0.0014	72.5
FP1 ($p=-1$)	843.75 (225)	3.75	$< 0.0001$	0.0043	73.3
FP1 ($p=2$)	1075.75 (225)	4.78	$< 0.0001$	$< 0.0001$	79.1
FP1 ($p=-2$)	1115.03 (225)	4.96	$< 0.0001$	$< 0.0001$	79.8
FP1 ($p=3$)	1156.48 (225)	5.14	$< 0.0001$	$< 0.0001$	80.5

**Fig. 3:**
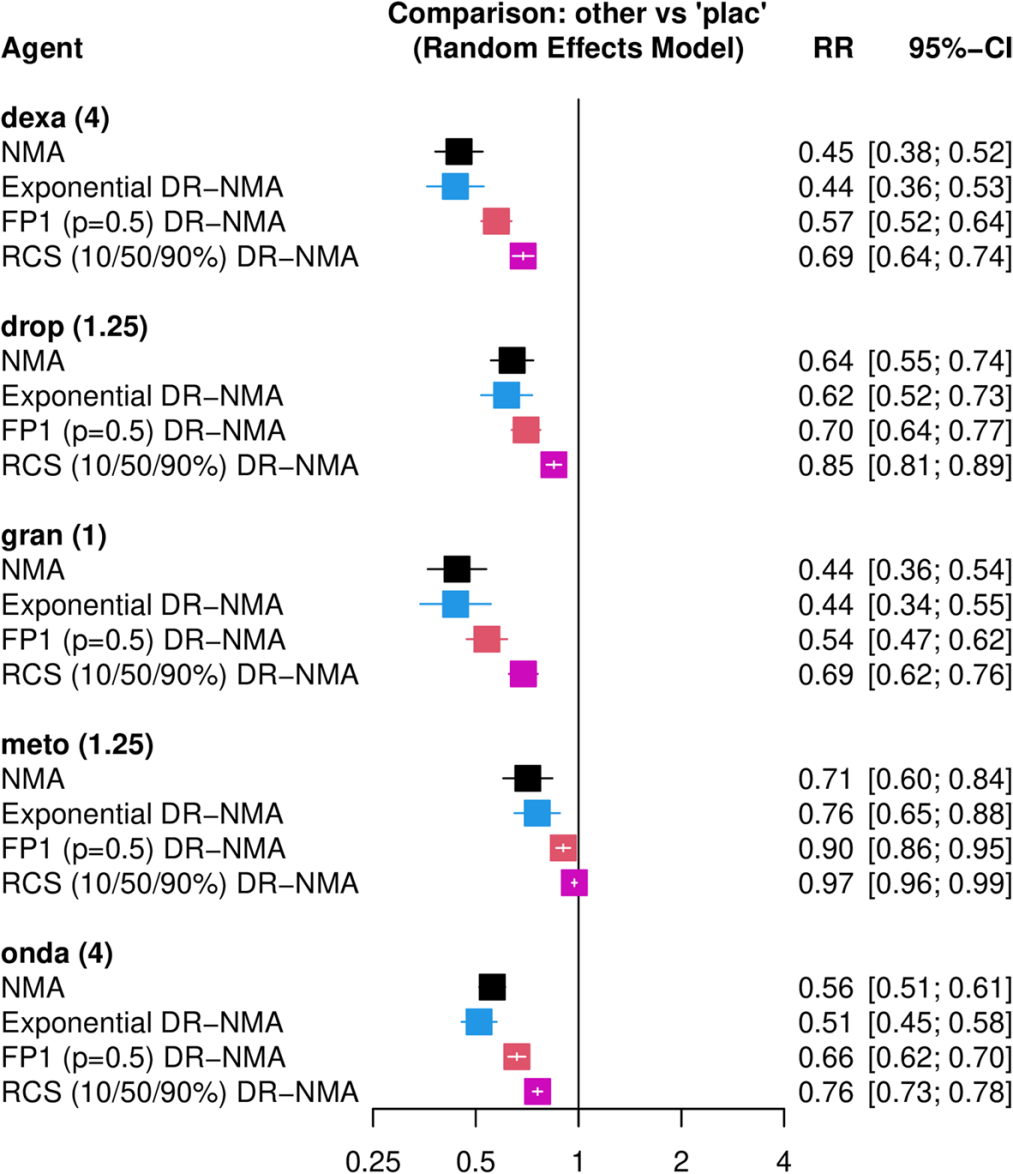
Forest plot for a subset of agents with their recommended doses (or the common dose, if a recommended dose is not available) indicated in parentheses. These doses are used in the DR-NMA models and ignored in the NMA model

Agent effects are depicted in a forest plot (figure in Additional file 2). The recommended dose of each agent is indicated in parentheses; when no recommended dose was available, we used the common dose, defined as the median dose observed for that specific agent across the included studies. Ondansetron (included in 132 comparisons), droperidol (56 comparisons), dexamethasone (51 comparisons), metoclopramide (44 comparisons), and granisetron (25 comparisons) were the five agents most commonly used in pairwise comparisons. A subset of the forest plot that features these five agents is shown in Fig. 3.

#### Standard NMA model

The standard NMA model exhibited substantial heterogeneity and inconsistency ($Q = 448.37$ with $df = 225$, $p\text {-value} < 0.0001$). The heterogeneity estimator is $\hat{\tau }^2 = 0.0612$ with $I^2 = 49.8\%$, suggesting considerable between-study heterogeneity (Table 2). The NMA model provides the best balance between fit and parsimony ($Q/df = 1.99$). The effects of the agents expressed as RR ($95\%$ CI) ranged from 0.06 [0.02, 0.21] for fosaprepitant to 1.08 [0.54, 2.15] for buspirone (Additional file 2). According to the standard NMA model, most drugs were found to have greater efficacy than placebo.

#### Exponential DR-NMA model

The exponential DR-NMA model revealed substantial heterogeneity and inconsistency ($Q_{d} = 475.02$, $df_{d} = 225$, $p\text {-value} < 0.0001$), with a heterogeneity estimate of $\hat{\tau }^2 = 0.1578$ and $I^2 = 52.6\%$ (Table 2). Among all DR-NMA models applied, the exponential model provided the best balance between model fit and complexity, with $Q_{d}/df_d = 2.11$, which was comparable to that of the standard NMA model.

For most of the agents, the estimated agent effects were closely aligned with those from the standard NMA model, leading to similar conclusions (Fig. 3, Additional file 2). Agents such as ondansetron and droperidol showed greater efficacy than placebo under the exponential DR-NMA model. As expected from the exponential model, the estimated dose-response curves for the most commonly used agents showed increasing efficacy with increasing dose, followed by a plateau (Additional file 3).

#### FP1 DR-NMA models

Among the FP1 dose-response models applied to the data, the model with power $p = 0.5$ provided the best balance between model fit and complexity, with $Q_{d}/df_d = 2.28$ (Table 2). However, its $Q_{d}/df_d$ was higher than that of the standard NMA and exponential DR-NMA models, suggesting comparatively poorer model adequacy. The heterogeneity estimate was $\hat{\tau }^2 = 0.0229$ with $I^2 = 56.1\%$, suggesting moderate heterogeneity. FP1 models with powers $p = -2, -1, 1, 2, \text {and}\ 3$ provided higher values of $Q_d / \textrm{df}_d$ and $I^2$, suggesting poorer model adequacy compared with the other DR-NMA models and the standard NMA.

**Fig. 4:**
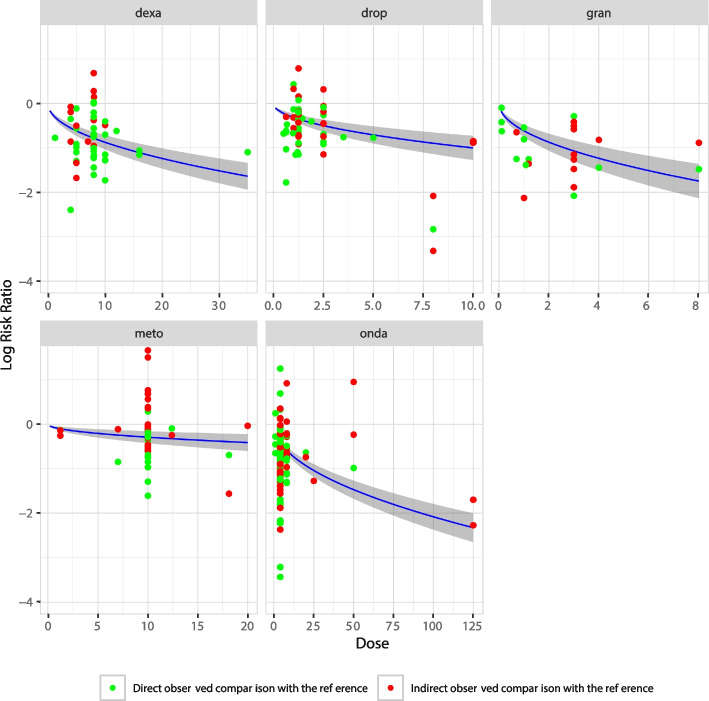
Dose-response curves for a DR-NMA with FP1 ($p=0.5$) for a subset of agents. The x-axis denotes the doses, while the y-axis denotes the log risk ratio from the comparison of two agents. The blue line represents the estimated dose-response curve from the FP1 ($p=0.5$) DR-NMA model, with a gray shadow indicating its $95\%$ confidence interval. The green points represent comparisons of an active agent with the reference agent (i.e., placebo), while the red points represent comparisons between two active agents. The indirect treatment effect is calculated by combining the observed effect of the first agent (relative to placebo) with the predicted effect of the second agent (relative to placebo), as derived from the DR-NMA model

For most agents, RRs with 95% CIs under the FP1 model with $p = 0.5$ were higher than, or comparable to, those from the standard NMA and exponential DR-NMA models (Fig. 3, Additional file 2). For the most commonly used agents, the FP1 model estimated lower efficacy compared with the NMA and exponential DR-NMA models (Fig. 3). Figure 4 presents the predicted dose-response relationships under FP1 with $p = 0.5$. Treatment efficacy increases with dose and varies across agents and dose levels.

#### RCS DR-NMA models

The RCS DR-NMA with three knots at the 10%, 50%, and 90% percentiles resulted in a poorer balance between fit and complexity ($Q_d/\textrm{df}_d = 2.76$) compared with the standard NMA, the exponential DR-NMA, or the FP1 ($p=0.5$) model. The estimated between-study variance was near zero ($\hat{\tau }^2 \approx 0$), as much of the variability was absorbed by the dose–response model, whereas $I^2$ remained relatively high (63.8%; Table 2). This behaviour arises because dose-response transformations rescale the treatment contrast, which can markedly affect the absolute heterogeneity estimate $\hat{\tau }^2$. By contrast, $I^2$ is a relative measure and is therefore less sensitive to such scale changes.

For 6 of the 26 agents ($\approx$23%) with few distinct dose levels, the RCS behaved nearly linearly, yielding identical point estimates across models but different confidence intervals. For agents with richer dose information (such as the most commonly used agents), RCS estimates tended to diverge from those of the NMA, exponential, and FP1 models by providing lower efficacy estimates, in some cases approaching the null or even exceeding it for certain agents (Fig. 3; Additional file 2). Additional file 4 presents dose–response plots that are non-monotonic, exhibiting an initial decrease in efficacy and a later increase. An alternative knot placement at the 25%/50%/75% percentiles led to similar conclusions (Table 2).

### Results of the antidepressant dataset

Additional file 5 presents an overview of the performance of the various (DR-)NMA models based on model adequacy and heterogeneity metrics. The FP1 model with power $p = 0$ ($Q_{d}/df_d = 1.74$) provided the best balance between model fit and parsimony, followed by FP1 with $p = 0.5$ ($Q_{d}/df_d = 1.76$) and the exponential model ($Q_{d}/df_d = 1.79$). The RCS with 10%, 50% and 90% percentiles resulted with 1.83. The standard NMA model yielded a higher ratio ($Q/df = 2.05$), while FP1 models with power $p = -2$ or $p = 3$ demonstrated poor fit.

#### Standard NMA model

The standard NMA model indicated substantial heterogeneity and inconsistency ($Q = 354.28$ with $df = 173$, $p\text {-value} < 0.0001$) (Additional file 5). The heterogeneity estimate is $\hat{\tau }^2 = 0.0681$ with $I^2 = 51.2\%$. The ratio $Q/df = 2.05$ was higher than for the alternative DR-NMA models — FP1 $(p = 0, 0.5, 1)$, exponential, and RCS — which exhibited a more favourable balance between fit and complexity. All antidepressants provided greater efficacy than placebo, with ORs [95% CIs] ranging from 1.12 [0.72, 1.75] for fluvoxamine to 1.83 [1.59, 2.10] for duloxetine (Additional file 6).

#### Exponential DR-NMA model

The exponential DR-NMA model revealed substantial heterogeneity and inconsistency ($Q_{d} = 477.23$, $df_{d} = 267$, $p\text {-value} < 0.0001$), with $\hat{\tau }^2 = 0.1218$ and $I^2 = 44.1\%$ (Additional file 5). The model provides a good balance between the model fit and complexity ($Q_{d}/df_d = 1.79$), and the dose-dependent effect estimates were comparable to those obtained from standard NMA (Additional file 6).

#### FP1 DR-NMA models

The FP1 model with $p = 0.5$ provided the second-best balance between model fit and complexity ($Q_{d}/df_d = 1.76$), following the FP1 model with $p = 0$, which showed the best performance (Additional file 5). Under the FP1 model with $p = 0.5$, the dose-dependent ORs indicated that all antidepressants were more effective than placebo (Additional file 6). Although the point estimates indicated lower efficacy compared to NMA, the narrower confidence intervals reflect greater precision (Additional file 6).

#### RCS DR-NMA models

The RCS DR-NMA model with knots at 10%, 50% and 90% percentiles provided a higher $Q_{d}/df_{d}$ ratio (value: $1.83$) compared with FP1 $(p = 0, 0.5)$ and exponential DR-NMA models. The flexible RCS dose–response function appears to absorb much of the variability and therefore drives the between-study variance $\tau ^2$ towards zero, whereas the corresponding relative measure of variability $I^2$ remains moderate (45.4%; Additional file 5). The forest plot (Additional file 6) indicates that most antidepressants were superior to placebo, with RCS model producing lower efficacy estimates relative to the other models. For some agents, such as clomipramine and reboxetine, the RCS model yielded forest-plot estimates suggesting no efficacy at the most commonly used doses. However, at higher doses the model indicated substantial efficacy, reflecting a non-linear dose–response pattern (Additional file 7). Analyses with alternative knot placements (25%, 50%, and 75% percentiles) yielded similar results (Additional file 5).

## Discussion

In this paper, we present a novel frequentist framework for DR-NMA that integrates flexible dose–response functions, such as exponential, fractional polynomials and restricted cubic splines, into the standard NMA model. These flexible models can improve the precision of DR-NMA when they effectively capture complex and nonlinear dose-response patterns.

A major advantage of our DR-NMA framework is its ability to address key limitations of conventional NMA methods, which overlook dose information through simplistic splitting or lumping strategies. By incorporating FP1 and RCS, our approach captures nonlinear dose-response relationships, offering nuanced insights into both the shape and magnitude of treatment effects across dose levels. This enables a more comprehensive evaluation of intervention performance, supporting informed clinical and policy decisions on the treatment efficacy and safety in diverse therapeutic settings.

The incorporation of flexible dose-response functions, such as FP1 and RCS, into DR-NMA enables the modeling of a wide range of dose-response patterns. FP1 offer a data-driven yet interpretable approach to capturing complex, nonlinear trends in treatment effects, while RCS provide a flexible framework for modeling intricate dose-effect relationships across interventions. DR-NMA models are particularly valuable in partially or fully disconnected networks, as they allow reconnection through shared doses, thereby enhancing the overall coherence of the network. By integrating these methods, DR-NMA improves the accuracy and robustness of dose-response estimation, even in complex or sparse evidence settings.

The frequentist formulation provides a transparent and accessible option for analysts who may be less familiar or comfortable with Bayesian inference, as it avoids the need for prior specification and relies on minimal modelling assumptions. Moreover, the approach is computationally efficient and readily implemented through the accompanying R package netdose, which facilitates the practical application of the proposed DR-NMA framework. An additional strength of the proposed DR-NMA framework is its ability to inform treatment optimisation by estimating treatment effects across a continuum of doses. By capturing nonlinear dose–response relationships, the models can help identify clinically relevant or potentially optimal dose levels, thereby supporting evidence synthesis and health technology assessment where dose selection plays a central role.

However, certain limitations of the method must be acknowledged. The results may be sensitive to the hypothesized dose-response relationship, underscoring the importance of selecting functions that align with plausible pharmacological or biological mechanisms. Additional care is needed when determining knot placement for the implementation of RCS. Sensitivity analyses — such as evaluating alternative dose-response functions or varying knot locations in RCS models — are essential for assessing robustness and ensuring the reliability of findings. While the existing literature suggests that knot placement is generally not a major concern [34, 36], simulation studies have shown that placing knots at points corresponding to sharp changes in effect may lead to more accurate and reliable estimates [15]. Moreover, our results highlight the importance of selecting models that are flexible enough to capture the underlying relationship, yet simple enough to remain stable and interpretable. We therefore encourage researchers to consider not only model fit but also model complexity and interpretability. Model adequacy statistics, such as the ratio $Q_d/df_d$, can help guide the selection of parsimonious models that are consistent with the data structure — ultimately supporting robust and interpretable conclusions. Future extensions of the framework could incorporate additional dose–response functions, such as Emax-type models. Although widely used in pharmacometrics, previous work has highlighted identifiability issues and challenges in sparse-dose or multi-arm settings, particularly when estimating Emax and ED$_{50}$ parameters [13]. Additionally, the proposed DR-NMA framework is designed to estimate dose–response relationships within the observed evidence and is not intended for forecasting the results of future trials.

Consistency in DR-NMA must be evaluated not only across direct and indirect evidence but also with respect to the chosen dose–response function. Recent work has extended inconsistency modelling to the dose–response setting in a Bayesian context using alternative functional forms [48]. Developing analogous inconsistency assessments within our frequentist DR-NMA framework represents an important avenue for future research. A further limitation is that we excluded multicomponent interventions and studies lacking dose information. Although this ensured methodological consistency, it may reduce the applicability of the approach to real-world datasets where composite interventions and incomplete dose reporting are common. Future work could extend component network meta-analysis (CNMA) to incorporate dose–response relationships, allowing the joint modelling of components and dose levels in multicomponent interventions.

This study lays the groundwork for advancing research on dose-response relationships within the context of frequentist network meta-analysis. Future directions include the development of hierarchical models to accommodate multi-level dose-response relationships or nested intervention effects, thereby further enhancing the flexibility and applicability of DR-NMA. Additionally, the integration of observational studies offers a promising avenue for validating dose-response relationships by broadening the evidence base and strengthening inferences through the synthesis of both randomized and nonrandomized data.

Our findings highlight important differences in model performance between datasets with sparse and rich dose structures. Sparse dose data — typically involving only two to three dose levels per agent — are common in NMAs based on RCTs, often due to ethical constraints, cost limitations, or variability in dose selection across studies. In contrast, richer dose structures — with seven to eight dose levels per agent — are more frequently encountered in systematic reviews or NMAs specifically designed to investigate detailed dose-response relationships. Such datasets are particularly common in fields like psychotherapy or chemotherapy, where a wide range of dose levels may be explored. By analyzing both types of datasets, we demonstrate that DR-NMA models are well-suited to accommodate the limitations of sparse data while also effectively capturing the detailed information available in richer dose networks, thereby supporting their broad applicability across diverse clinical research contexts.

Our findings emphasize the importance of selecting a dose–response model that aligns with the structure and richness of the available data. The examples presented highlight how different dose–response functions influence model fit, underscoring the need for careful model selection. While the RCS model offers high flexibility, in our applications it sometimes underestimated efficacy at commonly used doses for certain agents and produced non-monotonic dose–response patterns, with efficacy increasing only at higher doses. Such behavior, although consistent with the spline specification, may lead to clinically misleading interpretations. By contrast, simpler parametric models such as exponential or low-order FP1 functions provided more stable and interpretable estimates across both sparse and rich dose–response settings.

Further research, particularly through well-designed simulation studies, is essential to systematically evaluate the performance of various dose-response functions, including RCS and FP1, across different scenarios. Such studies can yield valuable insights into the specific strengths and limitations of each modeling approach, and thereby inform best practices for model selection. Although prior empirical comparisons of dose-response functions in meta-analyses have generally reported similar results across methods [49], more comprehensive evaluations across diverse settings are needed to clarify subtle differences in performance, robustness, and practical applicability.

The development of automated tools for dose-response model selection represents a promising avenue for future work. These tools could enhance reproducibility and efficiency by minimizing subjective decisions, streamlining the modeling process, and enabling more consistent identification of data-driven dose-response functions. Such innovations would facilitate broader adoption of DR-NMA in evidence synthesis.

## Conclusions

In this article, we propose a frequentist DR-NMA framework that enables the estimation of dose-dependent treatment effects and the prediction of efficacy or safety across a range of doses. The proposed methods are implemented in the R package netdose [19], facilitating their application in real-world analyses. Despite some limitations, the framework provides researchers, decision-makers, and regulatory bodies with a robust and flexible tool to support evidence synthesis, treatment optimization, and health technology assessment.

## Supplementary Information

Additional file 1. Annotated R code for DR-NMA analyses. Contains the full R script used to implement standard NMA and DR-NMA models (including linear, exponential, quadratic, FP1, and RCS) with the netdose package. The script includes applications to hypothetical data, the general anesthesia dataset, and the antidepressant dataset.

Additional file 2. Forest plot for general anesthesia dataset. Presents the complete forest plot of agent effects under the standard NMA, exponential, FP1 ($p=0.5$), and RCS model with knots at 10%, 50% and 90% percentiles. A subset is shown in Fig. 3.

Additional file 3. Dose–response plot using the exponential model for a subset of agents (general anesthesia).

Additional file 4. Dose–response plot using the RCS model with knots at 10%, 50% and 90% percentiles for a subset of agents (general anesthesia).

Additional file 5. Model fit statistics for antidepressant dataset.

Additional file 6. Forest plot for antidepressant dataset for all agent effects under the standard NMA, exponential, FP1 ($p=0.5$), and RCS model with knots at 10%, 50% and 90% percentiles.

Additional file 7. Dose–response plot using the RCS model with knots at 10%, 50% and 90% percentiles (antidepressant dataset).

## Data Availability

The anesthesia and antidepressant datasets used in this study are publicly available in the netdose R package, accessible at https://cran.r-project.org/package=netdose. All analyses can be reproduced using the R code provided in Additional file 1.

## Abbreviations

aliz: Alizapride
amis: Amisulpride
apre: Aprepitant
beta: Betamethasone
busp: Buspirone
cp12: CP-122,721
cycl: Cyclizine
dexa: Dexamethasone
dime: Dimenhydrinate
dixy: Dixyrazine
dola: Dolasetron
domp: Domperidone
drop: Droperidol
DR-NMA: Dose-response network meta-analysis
FP1: Fractional polynomial of order 1
fosa: Fosaprepitant
gran: Granisetron
halo: Haloperidol
mcg: Microgram
meto: Metoclopramide
NMA: Network meta-analysis
onda: Ondansetron
OR: Odds ratio
palo: Palonosetron
plac: Placebo
pred: Prednisone
proc: Prochlorperazine
prom: Promethazine
ramo: Ramosetron
RCS: Restricted cubic splines
rola: Rolapitant
RR: Risk ratio
scop: Scopolamine
trop: Tropisetron

## References

[1] Higgins J, Thomas J, Chandler J, Cumpston M, Li T, Page M, et al. Cochrane Handbook for Systematic Reviews of Interventions. 2nd ed. Chichester (UK): Wiley; 2019.

[2] Salanti G. Indirect and mixed-treatment comparison, network, or multiple-treatments meta-analysis: many names, many benefits, many concerns for the next generation evidence synthesis tool. Res Synth Methods. 2012;3(2):80–97. 10.1002/jrsm.1037. 26062083 10.1002/jrsm.1037

[3] Higgins J, Jackson D, Barrett J, Lu G, Ades A, White I. Consistency and inconsistency in network meta-analysis: concepts and models for multi-arm studies. Res Synth Methods. 2012;3(2):98–110. 10.1002/jrsm.1044. 26062084 10.1002/jrsm.1044 PMC4433772

[4] Petropoulou M, Nikolakopoulou A, Veroniki AA, Rios P, Vafaei A, Zarin W, et al. Bibliographic study showed improving statistical methodology of network meta-analyses published between 1999 and 2015. J Clin Epidemiol. 2017;82:20–8. 10.1016/j.jclinepi.2016.11.002. 27864068 10.1016/j.jclinepi.2016.11.002

[5] Greenland S, Longnecker MP. Methods for trend estimation from summarized dose-response data, with applications to meta-analysis. Am J Epidemiol. 1992;135(11):1301–9. 10.1093/oxfordjournals.aje.a116237. 1626547 10.1093/oxfordjournals.aje.a116237

[6] Hamling J, Lee P, Weitkunat R, Ambühl M. Facilitating meta-analyses by deriving relative effect and precision estimates for alternative comparisons from a set of estimates presented by exposure level or disease category. Stat Med. 2008;27(7):954–70. 10.1002/sim.3013. 17676579 10.1002/sim.3013

[7] Orsini N, Li R, Wolk A, Khudyakov P, Spiegelman D. Meta-analysis for linear and nonlinear dose-response relations: examples, an evaluation of approximations, and software. Am J Epidemiol. 2012;175(1):66–73. 10.1093/aje/kwr265. 22135359 10.1093/aje/kwr265 PMC3244608

[8] Crippa A, Orsini N. Dose-response meta-analysis of differences in means. BMC Med Res Methodol Vol. 2016;16:91. 10.1186/s12874-016-0189-0. 10.1186/s12874-016-0189-0 PMC4971698 27485429

[9] Crippa A, Orsini N. Multivariate Dose-response Meta-analysis: The dosresmeta R package. J Stat Softw Code Snippets. 2016;72(1):1–15. 10.18637/jss.v072.c01.

[10] Xu C, Doi SAR. The robust error meta-regression method for dose-response meta-analysis. Int J Evid Based Healthc. 2018;16(3):138–44. 10.1097/XEB.0000000000000132. 29251651 10.1097/XEB.0000000000000132

[11] Crippa A, Discacciati A, Bottai M, Spiegelman D, Orsini N. One-stage dose-response meta-analysis for aggregated data. Stat Methods Med Res. 2019;28(5):1579–96. 10.1177/0962280218773122. 29742975 10.1177/0962280218773122

[12] Mandema JW, Cox E, Alderman J. Therapeutic benefit of eletriptan compared to sumatriptan for the acute relief of migraine pain-results of a model-based meta-analysis that accounts for encapsulation. Cephalalgia. 2005;25:715–25. 10.1111/j.1468-2982.2004.00939.x. 16109054 10.1111/j.1468-2982.2004.00939.x

[13] Mawdsley D, Bennetts M, Dias S, Boucher M, Welton N. Model-Based Network Meta-Analysis: A Framework for Evidence Synthesis of Clinical Trial Data. CPT Pharmacometrics Syst Pharmacol. 2016;5(8):393–401. 10.1002/psp4.12091. 27479782 10.1002/psp4.12091 PMC4999602

[14] Pedder H, Dias S, Bennetts M, Boucher M, Welton N. Modelling time-course relationships with multiple treatments: Model-Based Network Meta-Analysis for continuous summary outcomes. Res Synth Methods. 2019;10(2):267–86. 10.1002/jrsm.1351. 31013000 10.1002/jrsm.1351 PMC6563489

[15] Hamza T, Cipriani A, Furukawa TA, Egger M, Orsini N, Salanti G. A Bayesian dose-response meta-analysis model: A simulations study and application. Stat Methods Med Res. 2021;30(5):1358–72. 10.1177/0962280220982643. 33504274 10.1177/0962280220982643 PMC8209313

[16] Hamza T, Furukawa TA, Orsini N, Cipriani A, Iglesias CP, Salanti G. A dose-effect network meta-analysis model with application in antidepressants using restricted cubic splines. Stat Methods Med Res. 2024;33(8):1461–72. 10.1177/09622802211070256. 35200062 10.1177/09622802211070256 PMC11462779

[17] Pedder H, Dias S, Bennetts M, Boucher M, Welton NJ. Joining the Dots: Linking Disconnected Networks of Evidence Using Dose-Response Model-Based Network Meta-Analysis. Med Decis Making. 2021;41(2):194–208. 10.1177/0272989X20983315. 33448252 10.1177/0272989X20983315 PMC7879230

[18] Sauerbrei W, Royston P. Investigating treatment-effect modification by a continuous covariate in IPD meta-analysis: an approach using fractional polynomials. BMC Med Res Methodol. 2022;22(1):98. 10.1186/s12874-022-01516-w. 35382744 10.1186/s12874-022-01516-w PMC8985287

[19] Petropoulou M, Schwarzer G. netdose: Dose-Response Network Meta-Analysis in a Frequentist Way. R package version 0.7-2. [Accessed: 11 October 2025]. https://github.com/petropouloumaria/netdose.

[20] Weibel S, Rücker G, Eberhart LHJ, Pace N, Hartl HM, Jordan OL, et al. Drugs for preventing postoperative nausea and vomiting in adults after general anaesthesia: a network meta-analysis. Cochrane Database Syst Rev. 2020;(10). 10.1002/14651858.CD012859.pub2. 10.1002/14651858.CD012859.pub2 PMC8094506 33075160

[21] Weibel S, Schaefer MS, Raj D, Rücker G, Pace NL, Schlesinger T, et al. Drugs for preventing postoperative nausea and vomiting in adults after general anaesthesia: an abridged Cochrane network meta-analysis. Anaesthesia. 2021;76:962–73. 10.1111/anae.15295. 33170514 10.1111/anae.15295

[22] Cipriani A, et al. Comparative efficacy and acceptability of 21 antidepressant drugs for the acute treatment of adults with major depressive disorder: a systematic review and network meta-analysis. Lancet. 2018;391:1357–66. 10.1016/S0140-6736(17)32802-7. 29477251 10.1016/S0140-6736(17)32802-7 PMC5889788

[23] Furukawa TA, Cipriani A, Cowen PJ, et al. Optimal dose of selective serotonin reuptake inhibitors, venlafaxine, and mirtazapine in major depression: a systematic review and dose-response meta-analysis. Lancet Psychiatry. 2019;6:601–9. 10.1016/S2215-0366(19)30188-0. 31178367 10.1016/S2215-0366(19)30217-2 PMC6586944

[24] Hamza T, Cipriani A, Furukawa TA, et al. A Bayesian dose-response meta-analysis model: A simulation study and application. Stat Methods Med Res. 2021. 10.1177/0962280219885713. 10.1177/0962280220982643 PMC8209313 33504274

[25] Hamza T, Furukawa TA, Orsini N, Cipriani A, Iglesias CP, Salanti G. A dose-effect network meta-analysis model with application in antidepressants using restricted cubic splines. Stat Methods Med Res. 2024;33(8):1461–72. 10.1177/09622802211070256. 35200062 10.1177/09622802211070256 PMC11462779

[26] Rücker G. Network meta-analysis, electrical networks and graph theory. Res Synth Methods. 2012;3(4):312–24. 10.1002/jrsm.1058. 26053424 10.1002/jrsm.1058

[27] Rücker G, Schwarzer G. Reduce dimension or reduce weights? Comparing two approaches to multi-arm studies in network meta-analysis. Stat Med. 2014;33:4353–69. 10.1002/sim.6236. 24942211 10.1002/sim.6236

[28] Seber GAF, Wild CJ. Nonlinear Regression. 1st ed. Hoboken: Wiley-Interscience; 2003.

[29] Royston P, Ambler G, Sauerbrei W. The use of fractional polynomials to model continuous risk variables in epidemiology. Int J Epidemiol. 1999;28(5):964–74. 10.1093/ije/28.5.964. 10597998 10.1093/ije/28.5.964

[30] Royston P, Sauerbrei W. Multivariable Model-Building: A Pragmatic Approach to Regression Analysis Based on Fractional Polynomials for Modelling Continuous Variables. Wiley Series in Probability and Statistics. Hoboken, NJ, USA: Wiley; 2008.

[31] Jansen J, Vieira M, Cope S. Network meta-analysis of longitudinal data using fractional polynomials. Stat Med. 2015;34(15):2294–311. 10.1002/sim.6492 25877808 10.1002/sim.6492

[32] Jansen JP. Network meta-analysis of survival data with fractional polynomials. BMC Med Res Methodol. 2011;11(61). 10.1186/1471-2288-11-61. 10.1186/1471-2288-11-61 PMC3112194 21548941

[33] Royston P. A strategy for modelling the effect of a continuous covariate in medicine and epidemiology. Stat Med. 2000;19(14):1831–47. 10.1002/1097-0258(20000730)19:14%3C;1831::aid-sim502%3E;3.0.co;2-1. 10867674 10.1002/1097-0258(20000730)19:14<1831::aid-sim502>3.0.co;2-1

[34] Harrell F. Regression Modeling Strategies: With Applications to Linear Models, Logistic and Ordinal Regression, and Survival Analysis. 2nd ed. Springer Series in Statistics. New York, NY, USA: Springer; 2015.

[35] Harrell F. Hmisc: Harrell Miscellaneous. R package version 5.2-4. [Accessed: 10 October 2025]. https://hbiostat.org/R/Hmisc/.

[36] Cj S. Comment: Generalized additive models. Stat Sci. 1986;1(3):312–4. 10.1214/ss/1177013605.

[37] Jackson D, White IR, Riley RD. Quantifying the impact of between-study heterogeneity in multivariate meta-analyses. Stat Med. 2012;31(29):3805–20. 10.1002/sim.5453. 22763950 10.1002/sim.5453 PMC3546377

[38] Jenkins DA, Martina R, Dequen P, Bujkiewicz S, Abrams K. The added value of real-world evidence to connect disconnected networks for network meta-analysis: a case study in rheumatoid arthritis. Value Health. 2016;19(7):A393. 10.1016/j.jval.2016.09.263.

[39] Goring S, Gustafson P, Liu Y, Saab S, Cline S, Platt R. Disconnected by design: Analytic approach in treatment networks having no common comparator. Res Synth Methods. 2016;7(4):420–32. 10.1002/jrsm.1204. 27061025 10.1002/jrsm.1204

[40] Phillippo D, Ades A, Dias S, Palmer S, Abrams K, Welton N. Methods for population-adjusted indirect comparisons in health technology appraisal. Med Decis Making. 2018;38(2):200–11. 10.1177/0272989X17725740. 28823204 10.1177/0272989X17725740 PMC5774635

[41] Signorovitch JE, Sikirica V, Erder MH, Xie J, Lu M, Hodgkins PS, et al. Matching-adjusted indirect comparisons: a new tool for timely comparative effectiveness research. Value Health. 2012;15(6):940–7. 10.1016/j.jval.2012.05.004. 22999145 10.1016/j.jval.2012.05.004

[42] Caro JJ, Ishak KJ. No head-to-head trial? simulate the missing arms. Pharmacoeconomics. 2010;28(10):957–67. 10.2165/11537420-000000000-00000. 20831304 10.2165/11537420-000000000-00000

[43] Thom HH, Capkun G, Cerulli A, Nixon RM, Howard LS. Network meta-analysis combining individual patient and aggregate data from a mixture of study designs with an application to pulmonary arterial hypertension. BMC Med Res Methodol. 2015;15(34). 10.1186/s12874-015-0007-0. 10.1186/s12874-015-0007-0 PMC4403724 25887646

[44] Rücker G, Schmitz S, Schwarzer G. Component network meta-analysis compared to a matching method in a disconnected network: A case study. Biom J. 2021;63(2):447–461. Epub 2020 Jun 28. 10.1002/bimj.201900339. 10.1002/bimj.201900339 32596834

[45] Petropoulou M, Rücker G, Weibel S, Kranke P, Schwarzer G. Model selection for component network meta-analysis in connected and disconnected networks: a simulation study. BMC Med Res Methodol. 2023;23(1):140. 10.1186/s12874-023-01959-9. 37316775 10.1186/s12874-023-01959-9 PMC10268445

[46] Welton NJ, Caldwell DM, Adamopoulos E, Vedhara K. Mixed treatment comparison meta-analysis of complex interventions: psychological interventions in coronary heart disease. Am J Epidemiol. 2009;169(9):1158–65. 10.1093/aje/kwp014. 19258485 10.1093/aje/kwp014

[47] Petropoulou M, Efthimiou O, Rücker G, Schwarzer G, Furukawa TA, Pompoli A, et al. A review of methods for addressing components of interventions in meta-analysis. PLoS ONE. 2021;16(2):e0246631. 10.1371/journal.pone.0246631. 33556155 10.1371/journal.pone.0246631 PMC7870082

[48] Pedder H, Dias S, Boucher M, Bennetts M, Mawdsley D, Welton NJ. Methods to assess evidence consistency in dose-response model based network meta-analysis. Stat Med. 2022;41(4):625–44. 10.1002/sim.9270. 34866221 10.1002/sim.9270

[49] Zhang C, Jia P, Yu L, Xu C. Introduction to methodology of dose-response meta-analysis for binary outcome: With application on software. J Evid Based Med. 2018;11:125–9. 10.1111/jebm.12267. 29345107 10.1111/jebm.12267

