# Supplementary figures and images for: Network meta-analysis with dose-response relationships
Source: BMC Med Res Methodol. 2026 Jan 13;26:17. doi: 10.1186/s12874-025-02754-4 (PMC12853944; doi:10.1186/s12874-025-02754-4)

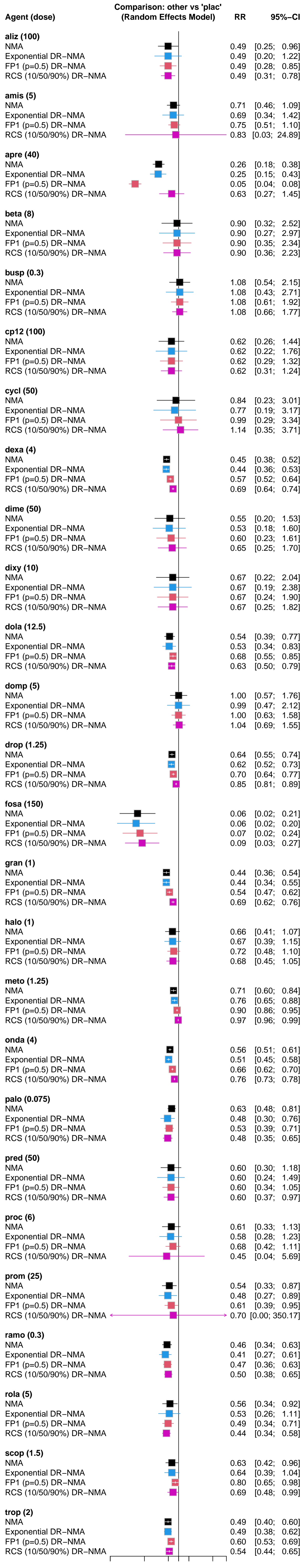

Supplement: Supplementary file 2 — Additional file 2. Forest plot for general anesthesia dataset. Presents the complete forest plot of agent effects under the standard NMA, exponential, FP1 ($p=0.5$), and RCS model with knots at 10%, 50% and 90% percentiles. A subset is shown in Fig. 3. [file 12874_2025_2754_MOESM2_ESM.pdf]

Log Risk Ratio

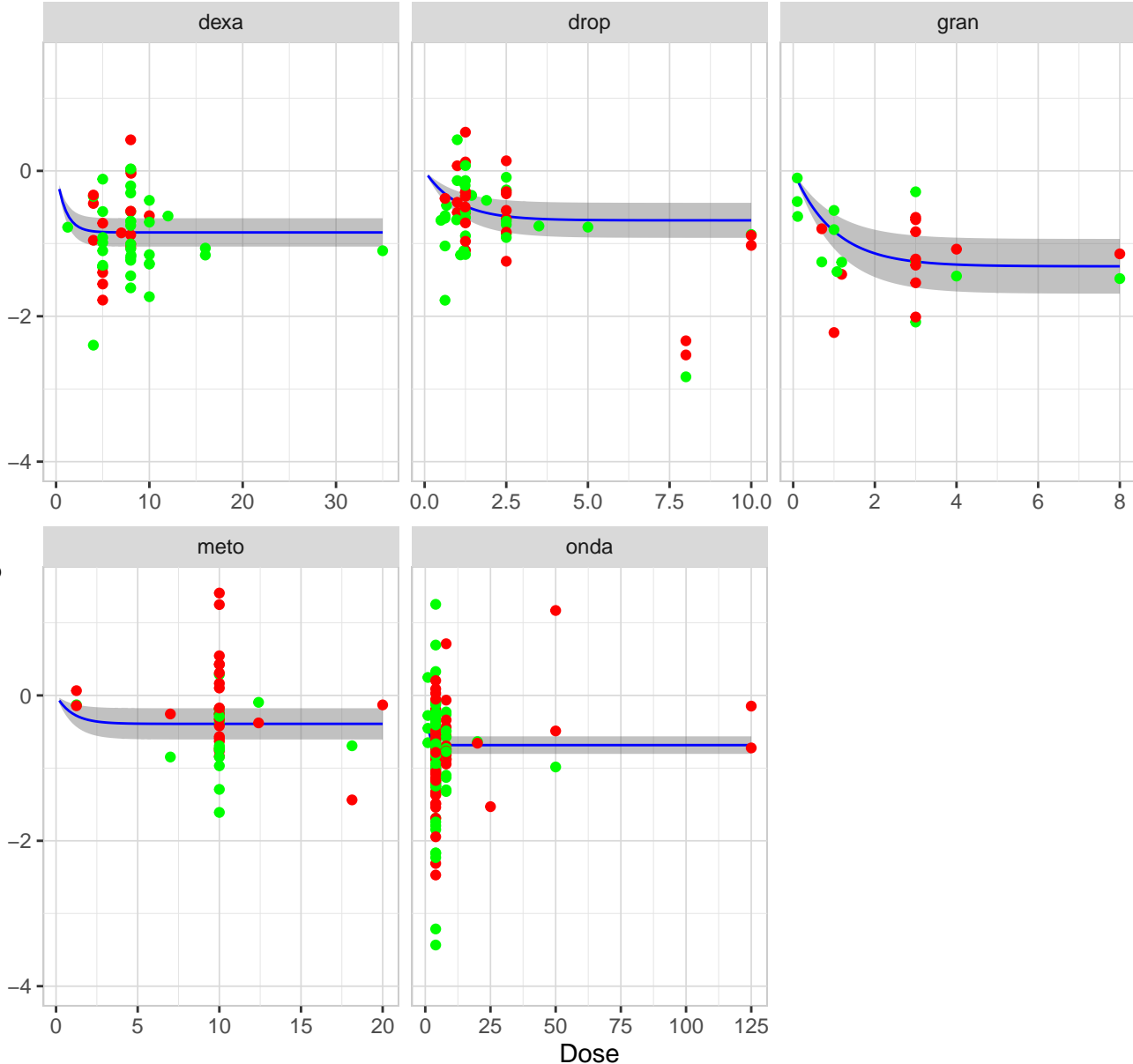

Supplement: Supplementary file 3 — Additional file 3. Dose–response plot using the exponential model for a subset of agents (general anesthesia). [file 12874_2025_2754_MOESM3_ESM.pdf]

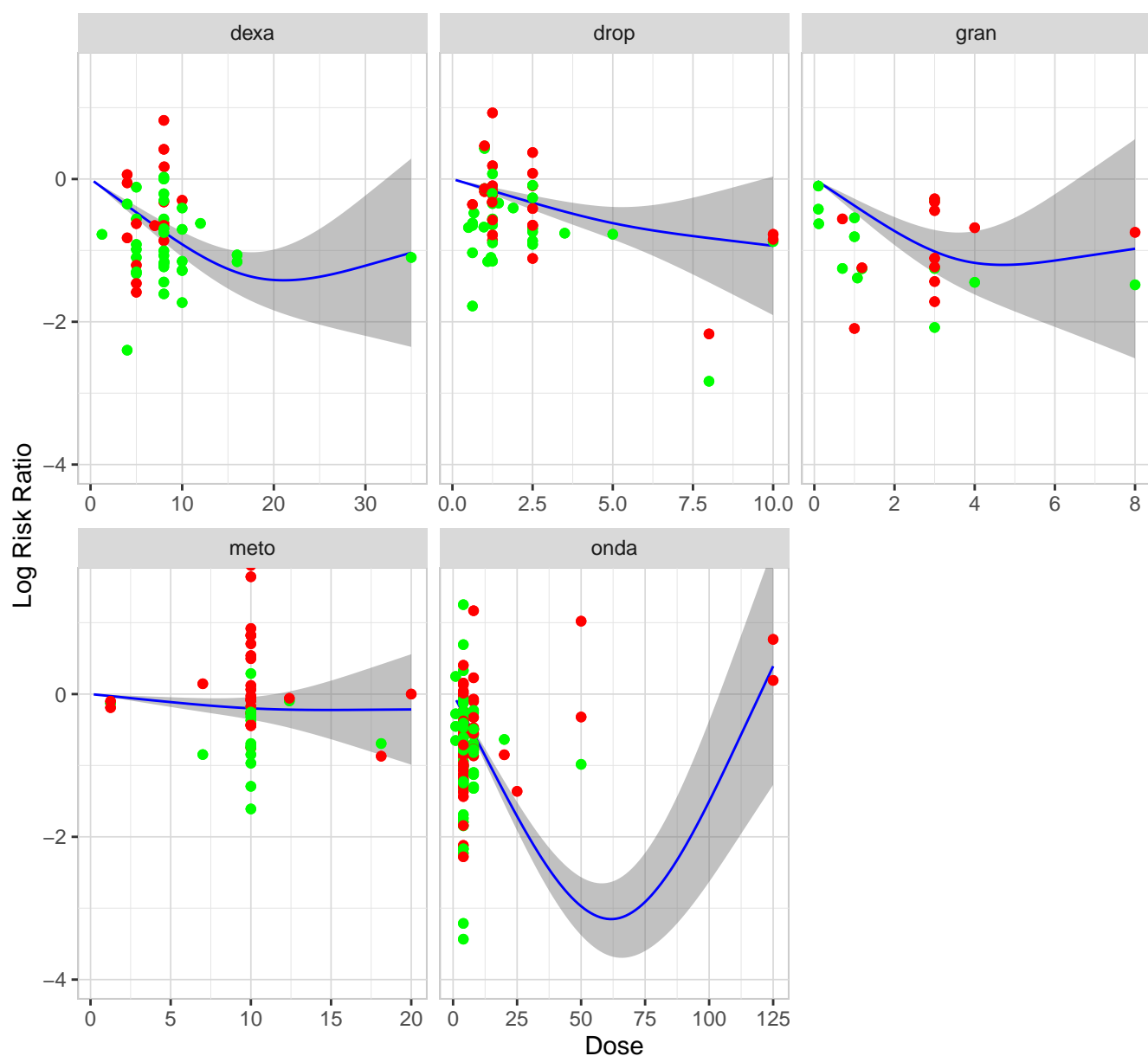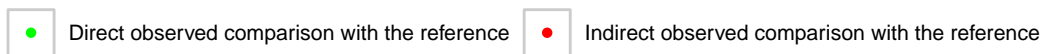

Supplement: Supplementary file 4 — Additional file 4. Dose–response plot using the RCS model with knots at 10%, 50% and 90% percentiles for a subset of agents (general anesthesia). [file 12874_2025_2754_MOESM4_ESM.pdf]

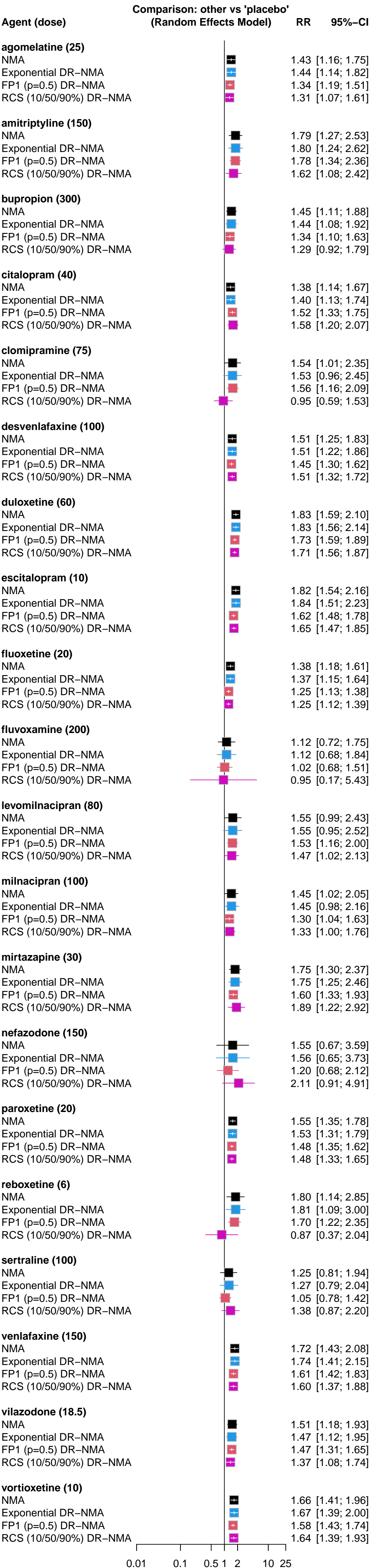

Supplement: Supplementary file 6 — Additional file 6. Forest plot for antidepressant dataset for all agent effects under the standard NMA, exponential, FP1 ($p=0.5$), and RCS model with knots at 10%, 50% and 90% percentiles. [file 12874_2025_2754_MOESM6_ESM.pdf]
