# Supplementary material for: Network meta-analysis with dose-response relationships
Source: BMC Med Res Methodol. 2026 Jan 13;26:17. doi: 10.1186/s12874-025-02754-4 (PMC12853944; doi:10.1186/s12874-025-02754-4)
Supplement: Supplementary file 5 — Additional file 5. Model fit statistics for antidepressant dataset. [file 12874_2025_2754_MOESM5_ESM.pdf]

(DR-)NMA ratio and heterogeneity metrics for antidepressant dataset (sorted by Q_d/df_d).

Model	Q_d (df_d)	Q_d/df_d	p-value(Q_d)	$\hat{\tau}^2$	I^2 (%)
FP1 ($p = 0$)	464.95 (267)	1.74	< 0.0001	0.0085	42.6
FP1 ($p = 0.5$)	470.87 (267)	1.76	< 0.0001	0.0021	43.3
Exponential	477.23 (267)	1.79	< 0.0001	0.1218	44.1
RCS (10/50/90%)	452.09 (247)	1.83	< 0.0001	< 0.0001	45.4
RCS (25/50/75%)	456.01 (247)	1.85	< 0.0001	< 0.0001	45.8
Linear/FP1 ($p = 1$)	536.41 (267)	2.01	< 0.0001	0.0000	50.2
NMA	354.28 (173)	2.05	< 0.0001	0.0681	51.2
Quadratic	544.64 (247)	2.21	< 0.0001	0.0000	54.6
FP1 ($p = -0.5$)	604.51 (267)	2.26	< 0.0001	3.6565	55.8
FP1 ($p = 2$)	651.82 (267)	2.44	< 0.0001	0.0000	59.0
FP1 ($p = -1$)	734.64 (267)	2.75	< 0.0001	32.8780	63.7
FP1 ($p = 3$)	767.43 (267)	2.87	< 0.0001	0.0000	65.2
FP1 ($p = -2$)	820.39 (267)	3.07	< 0.0001	372.3557	67.5
