# Supplementary material for: Network meta-analysis with dose-response relationships
Source: BMC Med Res Methodol. 2026 Jan 13;26:17. doi: 10.1186/s12874-025-02754-4 (PMC12853944; doi:10.1186/s12874-025-02754-4)
Supplement: Supplementary file 7 — Additional file 7. Dose–response plot using the RCS model with knots at 10%, 50% and 90% percentiles (antidepressant dataset). [file 12874_2025_2754_MOESM7_ESM.pdf]

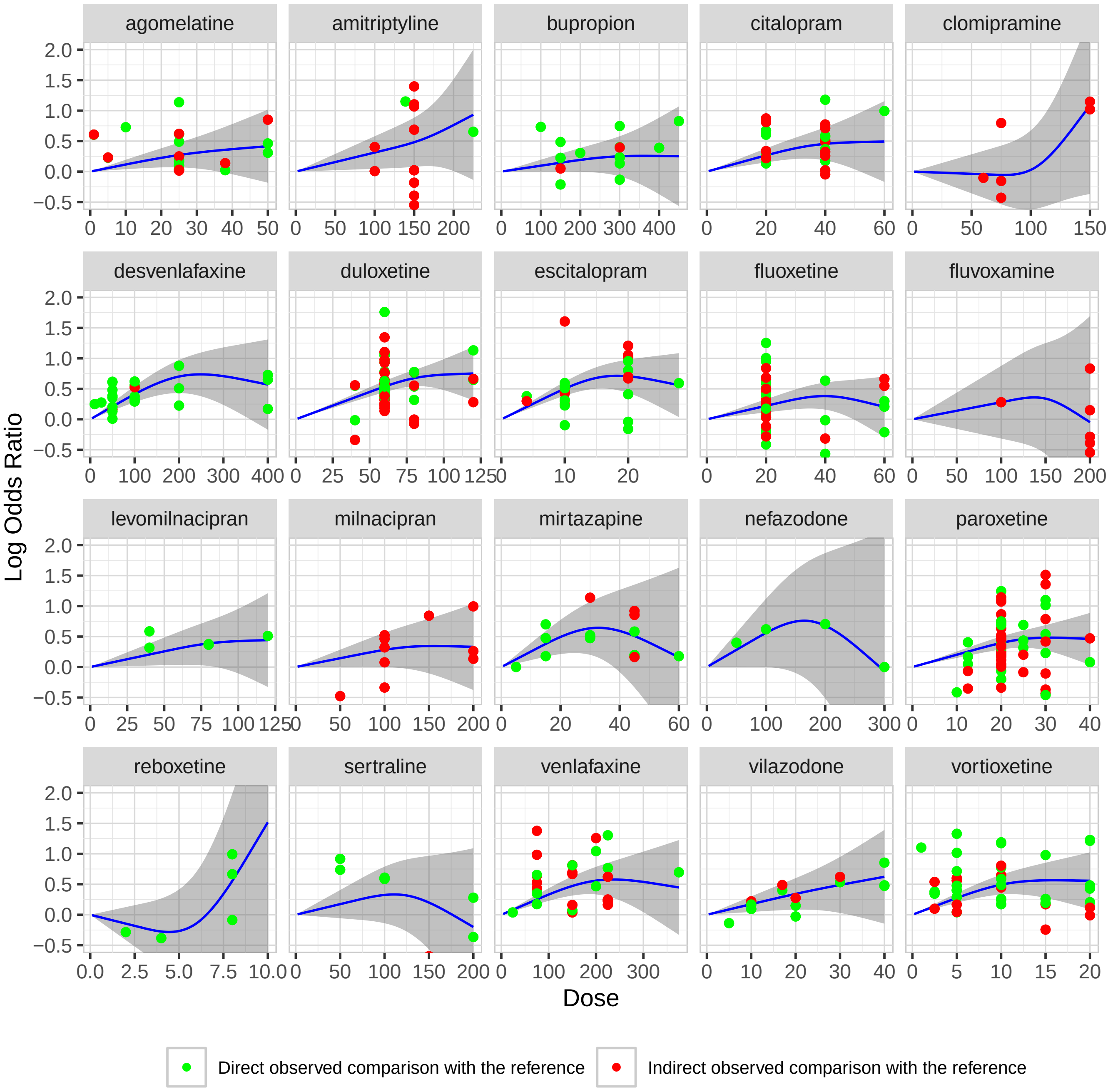

agomelatine
amitriptyline
bupropion
citalopram
clomipramine
desvenlafaxine
duloxetine
escitalopram
fluoxetine
fluvoxamine
levomilnacipran
milnacipran
mirtazapine
nefazodone
paroxetine
reboxetine
sertraline
venlafaxine
vilazodone
vortioxetine
Log Odds Ratio
Dose
Direct observed comparison with the reference
Indirect observed comparison with the reference
